# Soil stressors on ecophysiology of bauxite mine impacted soil: Heavy metal–acidity–organic matter nexus

**DOI:** 10.1002/jeq2.20666

**Published:** 2025-01-07

**Authors:** Kasturi Charan, Sonali Banerjee, Jajati Mandal, Pradip Bhattacharyya

**Affiliations:** ^1^ Agricultural and Ecological Research Unit Indian Statistical Institute Giridih Jharkhand India; ^2^ School of Science, Engineering & Environment University of Salford Manchester UK

## Abstract

Soil ecophysiology is adversely affected by various environmental hazards, particularly in mining regions. While there has been substantial research on the effects of coal, mica, copper (Cu), and manganese (Mn) mining on soil quality, the impact of bauxite mining operations on nearby soils has largely been overlooked in the literature. Therefore, this study aims to investigate how microbial activity and dynamics are influenced by soil stressors, such as acidity and heavy metals, in areas adjacent to active bauxite mines. Soil samples were collected from three adjacent locations of an active bauxite mine area at distances of <100 m (S1), 100–500 m (S2), and >500 m (S3). The samples contained chromium (Cr), copper (Cu), nickel (Ni), lead (Pb), zinc (Zn), manganese (Mn), and cadmium (Cd), as well as elevated acidity and aluminum (Al). These conditions adversely affected the soil microbial indicators, including fluorescein diacetate (FDA), microbial biomass carbon (MBC), and enzyme activity. The highest concentrations of labile metals (i.e., water‐soluble and exchangeable) were found in soil mixed with mining waste (S1), whereas acidity and Al were highest in sparsely vegetated soil (S3). Total acidity, total potential acidity, pH‐dependent acidity, and Al were significantly positively correlated. Moreover, the significant positive correlation among organic carbon (OC), acidity, Al, and microbial properties (FDA, MBC, and microbial enzymes) suggests a potential effect of OC in mitigating acidity in S3. The ratios of microbial properties with OC depicted a significant negative correlation with acidity and Al fraction, denoting that acidity and Al posed a deleterious effect on soil microbial health. The similarity percentage analysis identified acid phosphatase as the key enzyme accounting for ∼78% of the observed differences in enzyme composition across the sites. Visual MINTEQ modeling revealed that the sites were saturated with different Al‐bearing minerals. Pollution load index (PI) and the geo‐accumulation index (*I*_geo_) values identified the region as heavily contaminated (PI > 1). Finally, the health risk analysis revealed that Ni posed a potential carcinogenic risk for both adults and children.

## Core Ideas

- Bauxite mining practices addressed a high heavy metal load in surrounding soils.
- Labile fractions of the metals are the most important factor governing soil microbial quality.
- Synergistic stress caused by acidity and aluminum is effectively mitigated by soil organic matter.
- Dissolution‐precipitation dynamics revealed the presence of Cr‐, Pb‐, and Cd‐bearing minerals.
- Heavy metal contamination is responsible for carcinogenic and noncarcinogenic risk to humans.

**Abbreviations**
AMD: acid mine drainage
ANOSIM: analysis of similarity
AP: acid phosphatase
CBD: carbonate bound
CEC: cation exchange capacity
Ex: exchangeable
ExA: exchangeable acidity
Ex‐Al: exchangeable aluminum
Ext‐Al: extractable aluminum
FDA: fluorescein diacetate
GSD: glucosidase
HI: hazard index
HQ: hazard quotient
*I*_geo_
: geo‐accumulation index
MBC: microbial biomass carbon
MF: mobility factor
OC: organic carbon
OM: organic matter
ORG: organic bound
OXD: Fe and Mn oxide bound
PCA: principal component analysis
PDA: pH‐dependent acidity
PERMANOVA: permutational multivariate analysis of variance
PI: pollution load index
PTM: potentially toxic metal
qCO_2_
: metabolic quotient
ROS: reactive oxygen species
RQ: respiratory quotient
RS: residual fraction
SI: saturation index
SIMPER: similarity percentage
TA: total acidity
TCLP: toxicity characteristics leaching procedure
TOC: total organic carbon
TPA: total potential acidity
URS: urease
USEPA: United States Environmental Protection Agency
WS: water soluble

## 1 INTRODUCTION

Since the early 2000s, there has been a significant increase in mining activities worldwide to meet the demands of industry. The extraction of Al ore requires the mining of bauxite, which is a major industrial activity with global economic significance (Halder et al., 2024). Similar to other top bauxite‐producing countries such as Australia, China, Guinea, and Jamaica, open‐cast mining is widely practiced in India, where the resources are predominantly located on plateaus with flat tops that are 1–2 m below the surface (Lewis et al., 2012, 2010; X. Li et al., 2022). The disruption of soil caused by mining activities results in the destruction of the surface layer, a reduction of organic matter (OM), alterations in microbial species diversity, changes in soil composition, and loss of nutrients and habitat, all of which significantly impact the overall ecosystem (Harris & Omoregie, 2007; Yadav et al., 2022). Microorganisms, being one of the crucial parts of the biosphere, play an important role in ecosystem health. Soil, being a vital component of the terrestrial ecosystem, bears the largest reservoir of microbes and microbial diversity. Their health and activity are highly decisive for the functionality and productivity of soil and the well‐being of other ecosystem components (Chakraborty et al., 2024; Wahsha et al., 2017). Soil enzymes, organic carbon (OC) from microbial origin, and microbial respiration are the quantifiable attributes that give early warning of the deterioration of the soil quality. Many prior studies have revealed that intensive strip‐mining practices make it difficult to recover the soil quality of the bauxite mine surroundings (Melo et al., 2018; Oneț et al., 2022). Therefore, maintaining soil health in terms of microbial activity, nutritional richness, plant productivity, and restoration of degraded land and post‐mined areas is now a global concern and liability.

Soil acidity greatly influences the bioavailability of metals. Acid deposition due to mining activities is responsible for the reduction in soil pH, which acts as a key factor for metal mobility (Anza et al., 2021; Chakraborty et al., 2024). The source of soil acidity can come from acid mine drainage (AMD) generation, acid rain, or the intrinsic presence of Al^3+^ or H^+^ ions. Bauxite mining includes surface layer stripping, excavation, ore extraction, processing associated with waste rock dumps, and tailing deposition, leads to the generation of metallic pollutants and acidic mine seepage that leaves an adverse impression on the immediate environment (Kapusta & Sobczyk, 2015). There are reports of many environmental hazards around the world caused by open‐cast mining practices and improper management of the solid waste generated during mining (Mahar et al., 2016). The removal of vegetation before mining operations, elimination of surface organic materials, and degradation of soil structure are some of the implications of bauxite mining (Lewis et al., 2010). Additionally, previous studies reported several other impacts, including groundwater contamination, reduction in aquatic life, increased mobility of metals such as Al, Fe, Cd, and Mn, as well as elevated levels of suspended solids in water due to acidic mine seepage (Kamble, 2019). Moreover, the persistence of acidity and metal pollution may severely impact soil functioning. All of these have deleterious effects on the ecosystem as well as the general health of the local populace and mine workers.

Both soil acidity and metal poisoning independently put stress on soil microorganisms and soil biota. Moreover, the acidification of soil increases the mobility and bioavailability of metal, thereby creating a negative impact on soil ecophysiology (Adamczyk‐Szabela & Wolf, 2022; Kicińska et al., 2021). Although there are many reports on heavy metal stress or acidity stress on the ecosystem, the combined metal‐acidity effect on soil ecophysiological health and quality is yet to be explored. This is a stress‐on‐stress condition, where both factors can act in a synergistic or antagonistic way to each other. Numerous bioindicators and biomarkers have been proposed for a better understanding of soil quality (Bhaduri et al., 2022; Joos et al., 2023; Schloter et al., 2017). Even though the observation of bioindicator species produces biotic indices that more precisely depict the condition of the ecosystem, microbial parameters are still thought to be the most significant indicators since microbial communities respond to environmental changes the quickest (Anza et al., 2021; Ferrarini et al., 2014; Fontanetti et al., 2011). Reports on the impact of these stressors on soil functioning, particularly mine‐impacted soil, are scarce in the literature. The present study is an investigation of the impact of these stressors (acidity and heavy metals) on soil quality in the bauxite mine region. Moreover, the main goal was to monitor the state of soil microbial health in response to metal‐acidity stress and to examine the status of soil quality because of intensive mining practices, based on the assessment of soil microbial qualities. A field‐based study was undertaken in the bauxite mine area of Jharkhand, India, with the following objectives: (1) to assess the effect of different forms of acidity on soil microbial health; (2) to explore the metal distribution pattern across the study area; (3) to understand relationships among acidity, metal, and soil microbial activity; and (4) to assess the role of OM on microbial activity.

## 2 MATERIALS AND METHODS

### 2.1 Sampling and preparation

A total of 60 samples were collected from three different sites of the bauxite mining area (23.47° N to 84.62° E) situated at an elevation of 2100 ft in the Lohardaga district of Jharkhand state in India, at distances of <100, 100–500, and >500 m. From each site, 20 soil samples at a depth of 0–10 cm were collected from the upper horizon as recommended by Ferrarini et al. (2014) and Wahsha et al. (2017) and brought to the laboratory in a labeled and sealed polythene bag and kept below 4°C in the refrigerator for further analysis. The soil samples were designated as Site 1 or S1 (mining waste dumped soil), Site 2 or S2 (reclaimed site soil), and Site 3 or S3 (soil from the site with sparse vegetation). For physicochemical analysis, samples were air‐dried, sieved through 2 and 0.2 mm, and stored for further study.

**Core Ideas**

- Bauxite mining practices addressed a high heavy metal load in surrounding soils.
- Labile fractions of the metals are the most important factor governing soil microbial quality.
- Synergistic stress caused by acidity and aluminum is effectively mitigated by soil organic matter.
- Dissolution‐precipitation dynamics revealed the presence of Cr‐, Pb‐, and Cd‐bearing minerals.
- Heavy metal contamination is responsible for carcinogenic and noncarcinogenic risk to humans.

### 2.2 Physicochemical characterization of the soil

The physicochemical analyses were carried out using (2 mm and 0.2 mm) air‐dried samples. The pH (H_2_O) was measured in a 1:2.5 soil water suspension. Total organic carbon (TOC) was measured by taking 1 g of a 0.2‐mm sample according to the modified Walkley and Black (1965) method. The cation exchange capacity (CEC) was assessed by the method outlined by Harada and Inoko (1980). Mineralizable N was determined by using the standard alkaline permanganate protocol wherein the soil was mixed with alkaline KMnO_4_ solution, releasing nitrogen, which was determined via ammonia distillation. The distillate was then absorbed in standard 0.02 (N) H_2_SO_4_, and the excess acid (H_2_SO_4_) was back titrated with normal 0.01 (N) NaOH using the methyl red indicator (Page et al., 1982). Available P was measured following Bray's method (Bray & Kurtz, 1945). The sand, silt, and clay content of the soil was measured using the standard hydrometer method. Total acidity (TA) was determined by extracting the soil solution with 1(N) NaOAc (pH 8.2) solution and then titrating it with 0.1 (N) NaOH. Exchangeable acidity (ExA) was determined by taking 10 g of soil mixed with 50 mL of 1 (M) KCl solution and titrated with standardized 0.1 (M) NaOH using a phenolphthalein indicator (McLean, 1983). To determine the exchangeable aluminum (Ex‐Al), 0.1 (M) KF was added to the above‐titrated solution and again titrated against standardized 0.1 (M) HCl (Peech et al., 1962). Total potential acidity (TPA) was quantified by the method outlined by Peech et al. (1962), where 10 g of soil was leached with a solution of 0.5 N BaCl_2_ and triethanolamine buffered at pH 8.2. The resulting leachate was further titrated with 0.2 N HCl. The pH‐dependent acidity (PDA) was computed as the difference between TPA and ExA (Coleman & Thomas, 1967). The extractable aluminum (Ext‐Al) was assessed by extracting the soil with 1.0 M NH_4_OAc adjusted to pH 4.8 and measured spectrophotometrically at 535 nm. Sequential potentially toxic metal (PTM) extraction was carried out following Tessier et al. (1979), which is thoroughly discussed in the Supporting Information Section 1. Six different fractions, that is, water soluble (WS), exchangeable (Ex), carbonate bound (CBD), Fe and Mn oxide bound (OXD), organic bound (ORG), and residual fraction (RS) of various PTMs were extracted. Atomic absorption spectroscopy (Systronics AA S‐816) was used to quantify metal contents in soil extracts. To assure instrument accuracy, a blank, and one calibration standard were performed after every 10 samples. Calibration standards were prepared from a standard stock solution of 1000 ppm (Sigma‐Aldrich grade).

### 2.3 Microbial analyses

To carry out the microbiological parameters, the field moist sample was used. Soil respiration was quantified through the estimation of released CO_2_ trapped in NaOH when 10 gm of soil was incubated with or without 0.5% glucose in a closed system at 22°C for 5 h. However, the incubation period for basal respiration was 24 h. The NaOH was then titrated against 0.05 M HCl. Microbial biomass carbon (MBC) was determined by the fumigation extraction method (Tabatabai, 1994). Dehydrogenase (DHG) was measured according to the standard method given by Casida et al. (1964). The activity was assessed by estimating triphenyl formazan, which was produced by reducing triphenyl tetrazolium chloride after incubating 6 g of soil at 37°C for 24 h. Catalase was measured by the KMnO_4_ titration method following Tan et al. (2014). Fluorescein diacetate (FDA) hydrolysis activity was quantified spectrophotometrically when 1 g soil was incubated with sodium phosphate buffer (pH 7.6) and FDA solution at 25°C for 3 h. Acid phosphatase (AP), aryl sulfatase (AS), and glucosidase (GSD) activity in soils were measured following methods suggested by Tabatabai (1994). To describe this, 1 g of moist soil was treated with a modified universal buffer including p‐nitrophenyl phosphate, sulfate, or glucopyranoside for 1 h at 37°C. The released p‐nitrophenol was detected spectrophotometrically at 420 nm. Urease (URS) activity was determined by the buffer method, which involves quantification of NH_4_^+^ N that was released when 5 g of soil was incubated with tris(hydroxymethyl)aminomethane buffer at optimal pH 9 with or without urea solution at 37°C for 2 h. The microbial metabolic quotient (qCO_2_) and respiratory quotient (RQ) were calculated by using the values of MBC, basal respiration, and substrate‐induced respiration with the help of formulas (1) and (2) presented by Chakraborty et al. (2022).

(1) $$\begin{array}{ccc} & & \mathrm{Microbial}\;\mathrm{metabolic}\;\mathrm{quotient}\;\left(\mathrm{qC}O_{2}\right)\\ & & \;=\frac{CO_{2}\;-\;\mathrm{CC}\;\mathrm{from}\;\mathrm{basal}\;\mathrm{respiration}}{\mathrm{Microbial}\;\mathrm{biomass}\;C} \end{array}$$

(2) $$\mathrm{Respiratory}\;\mathrm{quotient}\;(\mathrm{RQ})=\frac{\mathrm{Basal}\;\mathrm{respiration}}{\mathrm{Substrate}\;\mathrm{induced}\;\mathrm{respiration}}$$

### 2.4 Ecological and human health hazard assessment

Table S1 provides the specific equation for each parameter pertaining to the assessment of ecological and health hazards. The cumulative impact of PTMs was evaluated by calculating the pollution load index (PI) using a formula presented by Banerjee et al. (2023). Furthermore, a geo‐accumulation index (*I*_geo_) was computed following Mondal et al. (2017) to assume metals' possible accumulation patterns in the upcoming period. The classification of contamination level based on *I*_geo_ is depicted in Table S2. Mobility of heavy metals in soil is a distinct attribute making eco‐toxicity in the environment and can be measured by calculating the mobility factor (MF) proposed by Kabala and Singh (2001). To determine the noncarcinogenic toxicity in human subjects, three exposure pathways, namely, intake ingestion, intake inhalation, and intake dermal, were computed and taken for calculating the hazard quotient (HQ) and hazard index (HI) (Mondal et al., 2017). The risk for significant non‐cancer health hazards is seemingly high when HI is >1. Cancer risk was computed by following the equation presented by USEPA (2002) and Banerjee et al. (2023).

### 2.5 Ions‐solubility assessment through visual MINTEQ model and toxicity characteristics leaching procedure assessment

The solubility experiment was carried out by following the method of Sahariah et al. (2015). Briefly, 10 g of dry soil sample was mixed with 100 mL of deionized water. The conical flasks were placed on a mechanical shaker at 120 rpm for 7, 14, and 21 days. At the completion of incubation, the soil suspension was filtered and analyzed for the respective cations (Na^+^, K^+^, Ca^2+^, and Mg^2+^), anions (NO_3_^−^, SO_4_^2−^, Cl^−^, and PO_4_^3−^), and PTMs (Pb, Ni, Cd, Cu, Cr, Fe, Zn, Mn, and Al). Mg^2+^ and other PTMs were quantified using atomic absorption spectroscopy (Systronics AA S‐816). Cations were quantified using a flame photometer (Systronics 130), and anions were measured by following the standard protocol discussed in the Supporting Information Section 2. The obtained data from 7, 14, and 21 days were analyzed through visual MINTEQ (4.0) (Gustafsson, 2023) geochemical modeling to derive the saturation index (SI). The model indicates that the ionic equilibrium of the solid‐phase matrix, supersaturation, and undersaturation is specified by SI values of 0, >0, and <0, respectively (Sahariah et al., 2015). The United States Environmental Protection Agency (USEPA) SW‐846 Method 1311 was used in the toxicity characteristics leaching procedure (TCLP) test to assess the toxicity of heavy metals. For TCLP, two types of extractants were used depending on the soil pH. When the soil pH was above 5, reagent 1 was used, containing 5.7 mL of CH_3_COOH diluted to 1 L. The extractant's pH was adjusted to 2.88 by adding 1 M HNO_3_ or 1 M NaOH. Similarly, when soil pH was <5, reagent 2 was employed, which was prepared by mixing 5.7 mL of CH_3_COOH with 64.3 mL of 1 M NaOH to achieve the pH value of 4.93. In the current investigation, both extractants were used based on soil pH. In the procedure, 2 g of soil was mixed with 40 mL of extractant and was shaken for 18 h at 30 ± 2 rpm. After 18 h of shaking, the soil extract was separated by centrifugation at 5000 rpm and tested for the presence of PTM using the Systronics AAS‐816.

### 2.6 Statistical analysis

Spearman correlation, Kruskal Wallis test, least significant difference post hoc analysis, two‐way analysis of variance (ANOVA), analysis of similarity (ANOSIM), permutational multivariate analysis of variance (PERMANOVA), principal component analysis (PCA), and cluster analysis were done by using the SPSS version 25 statistical software package.

## 3 RESULTS

### 3.1 Physicochemical characteristics of the study site

The overall physicochemical attributes are tabulated in Table S3. The pH across the study area was acidic, ranging from 4.02 to 4.9 (4.33 ± 0.30) at S1, 4.6 to 5.3 (5.03 ± 0.17) at S2, and 4.0 to 4.9 (4.54 ± 0.28) at S3. TOC and mineralizable N were significantly high at S3 ranging from 1.56% to 2.19% (1.93 ± 0.15%) and 0.007% to 0.01% (0.011 ± 0.005%), respectively (LSD_TOC _= 0.06; LSD_minN _= 0.001; *p* < 0.001). The OM content was highest at S3 (3.40 ± 0.27%). Corresponding to soil texture, the sand content was maximum in S1 (71.43 ± 9.55%) while clay was highest in S3 (35.56 ± 5.03%). Ex cations (Ca^2+^, K^+^, Mg^2+^, and Na^+^) were highest at S2. The CEC, another important attribute defining soil's chemical characteristics, was significantly high at S3 (24.45 ± 3.75 cmol kg^−1^), followed by S2 and S1 which exhibited lower CEC with a mean of 10.59 ± 1.51 and 5.42 ± 1.53 cmol kg^−1^, respectively (LSD = 0.79; *p* < 0.001). Available P was poor across sites.

### 3.2 Distribution of PTMs in different fractions: Sequential extraction‐based evaluation

**FIGURE 1:**
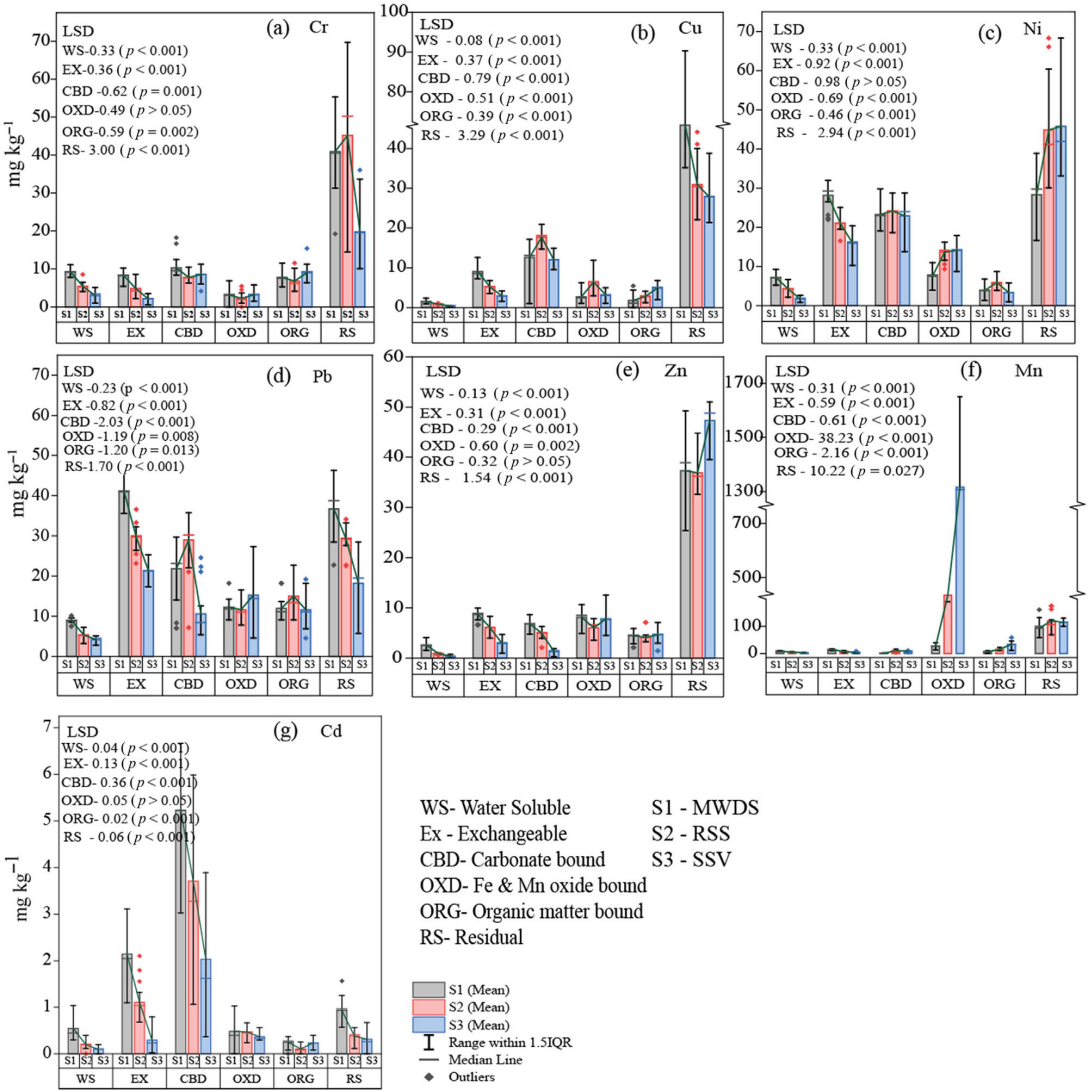
Fractional distribution of potentially toxic metals (PTMs) across the study area (S1: mining waste dumped soil [MWDS]; S2: reclaimed site soil [RSS]; and S3: soil from the site with sparse vegetation [SSV]). LSD, least significant difference.

The distribution of the sequentially extracted fractions of the studied PTMs is given in Figure 1. The highest concentration was found in the RS in Cr, Cu, Ni, and Zn, while the OXD fraction was highest in Mn and CBD in Cd. Pb was distributed in the Ex, CBD, OXD, ORG, and RS fractions in a relatively comparable manner. Although the insoluble forms of metals surpassed other fractions, the occurrence of WS and Ex was found to be significantly higher at S1 as compared to S2 and S3. Among all metals, the Ex fraction of Ni and Pb was high across the study area, that is, 10.25–31.96 mg kg^−1^ (approximately 15%–29%) and 17.27–46.36 mg kg^−1^ (approximately 26%–31%), respectively. Cr was largely distributed in the RS fraction and followed the order S2 > S1 > S3. Cu was predominantly observed in the RS fractions, followed by CBD, Ex, OXD, ORG, and WS fractions. The highest Cd was found in CBD and Ex fractions. The CBD‐Cd ranged between 0.36 and 6.66 mg kg^−1^ (approximately 55%–61%), while Ex‐Cd ranged from 0.02 to 3.1 mg kg^−1^ (approximately 9%–22%). Although Zn and Mn are not considered PTMs, their essentiality runs parallel to their toxicity when present in elevated amounts or with long‐term exposure. Most of the Zn was associated with the RS fraction, and the OXD fraction had the next highest amount of Zn associated with it. The RS‐bound zinc ranged from 25.36 to 51.05 mg kg⁻¹ (approximately 55%–73%), while the OXD‐bound fraction varied between 3.56 and 12.54 mg kg⁻¹ (approximately 10%–12%) across S1, S2, and S3. Mn was predominantly found in the OXD fraction (13.33–1650.96 mg kg^−1^; approximately 18%–89%) and followed the order of S3 > S2 > S1 (LSD for OXD‐Mn = 38.23; *p* < 0.001). The occurrence of Fe was extensive in the study area (Figure S1). Almost 18,796.87–238,009.01 mg kg^−1^ (approximately 76%–97%) of Fe was associated with RS‐bound fractions that are practically inert. Compared with this RS fraction of Fe, other fractions hold a very nominal part; only the OXD‐bound fraction occupied a little more, nearly 2033.96–12,957.10 mg kg^−1^ (approximately 3%–23%). Even if the RS‐Fe was highest in the S1 site (LSD‐Fe_RS _= 8131.02; *p* < 0.001), nevertheless, the CBD‐Fe, ORG‐Fe, and OXD‐Fe were highest in S3 (LSD‐Fe_CBD_ = 1.55, LSD‐Fe_ORG_ = 3.25, LSD‐Fe_OXD _= 365.16; *p* < 0.001). Considering the total concentration (i.e., summation of six fractions), the presence of PTM was more in S1, except Ni and Mn, which were highest in S2 and S3, respectively (Table S3).

### 3.3 Soil acidity and aluminum availability

**FIGURE 2:**
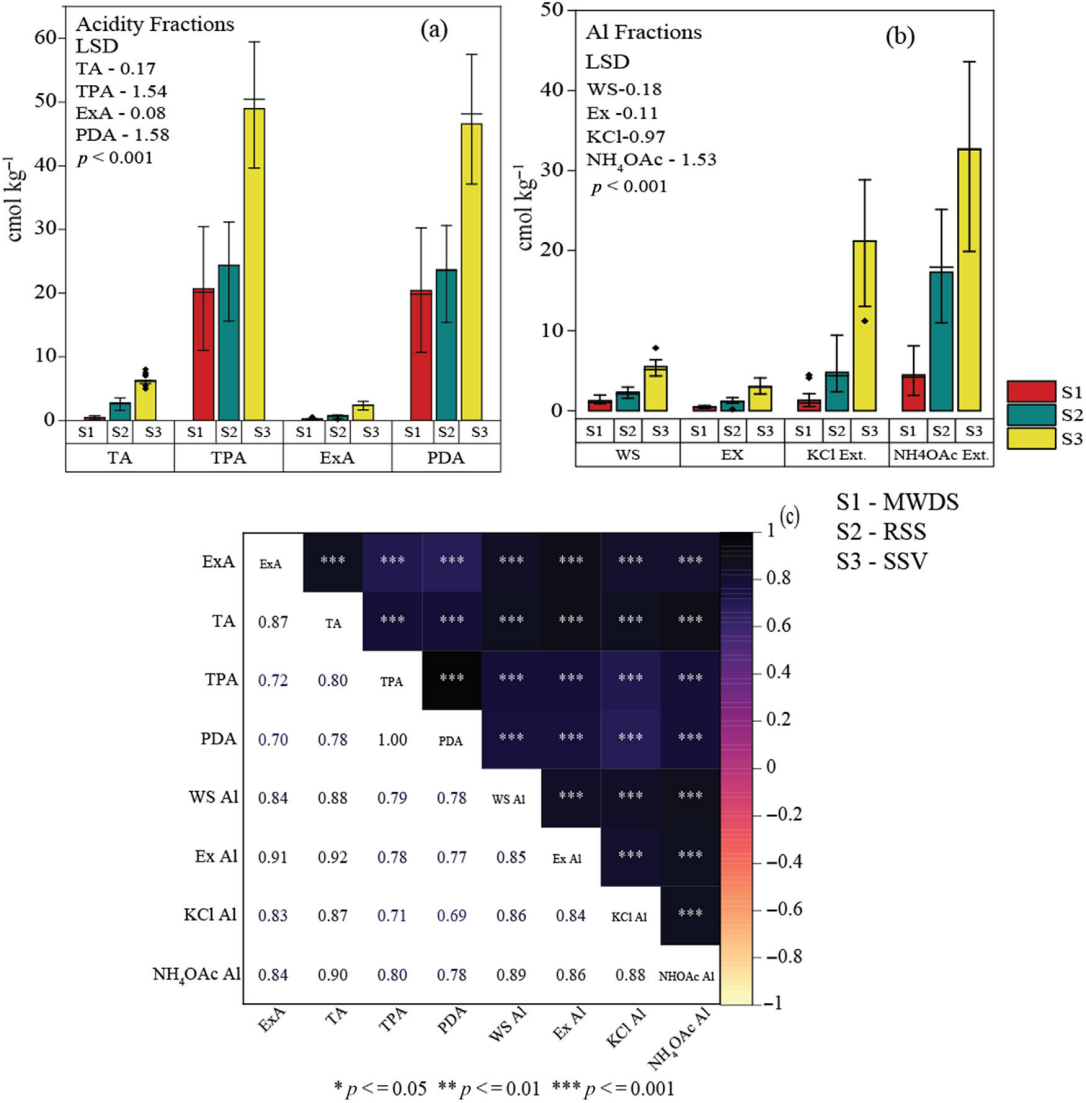
(a) Different fractions of acidity (b) Different fractions of aluminum (Al) (c) Correlation between acidity and aluminum (Al) (S1: mining waste dumped soil [MWDS]; S2: reclaimed site soil [RSS]; and S3: soil from the site with sparse vegetation [SSV]). EX, exchangeable; ExA, exchangeable acidity; ExAl, exchangeable aluminum; LSD, least significant difference; PDA, pH‐dependent acidity; TA, total acidity; TPA, total potential acidity; WS, water soluble.

TPA values ranged from 11.03 to 59.4 cmol kg^−1^ in the study area and were substantially high at S3 (48.94 ± 6.02 cmol kg^−1^), followed by S2 (24.32 ± 3.95 cmol kg^−1^) and S1 (20.68 ± 4.47 cmol kg^−1^) (Figure 2a). The PDA (deducted fraction of ExA from TPA) ranged from 10.72 to 57.46 cmol kg^−1^ across the sites, thus occupying a greater proportion toward TPA. Likewise, it was maximum at S3 (46.56 ± 6.27 cmol kg^−1^). TA varied from 0.20 to 8.02 cmol kg^−1^, while the ExA ranged from 0.2 to 3.01 cmol kg^−1^ across the study area, which was much less than TPA and PDA. TA and ExA were consistently high at the S3 (TA = 6.28 ± 0.69; ExA = 2.37 ± 0.43 cmol kg^−1^), followed by S2 (TA = 2.61 ± 0.63; ExA = 0.68 ± 0.13 cmol kg^−1^) and S1 (TA = 0.44 ± 0.13; ExA = 0.30 ± 0.10 cmol kg^−1^) (LSD_TA _= 0.17; LSD_ExA _= 0.08; *p* < 0.001). Ex‐Al contributed a significant part of ExA ranging from 0.29 to 0.65 (0.47 ± 0.10 cmol kg^−1^), 0.9 to 1.64 (1.17 ± 0.32 cmol kg^−1^), and 2.1 to 4.12 (3.01 ± 0.51 cmol kg^−1^), respectively, for S1, S2, and S3 (Figure 2b). ExA showed a highly significant positive correlation (*r* = 0.91, *p* < 0.001) with Ex‐Al inferring the dominant role of aluminum in making ExA (Figure 2c). KCl‐aluminon Ext‐Al varied from 0.53 to 28.87 cmol kg^−1^; however, the NH_4_OAc‐aluminon extraction ranged from 2.16 to 43.61 cmol kg^−1^. Figure 2b depicts the highest amount of Al was extracted from S3 soil (21.16 ± 4.74 KCl‐Al; 32.64 ± 7.0 NH_4_OAc‐Al) in both KCl and NH_4_OAc extractants, followed by S2 (4.83 ± 2.23 KCl; 17.30 ± 4.10 NH_4_OAc) and S1 (1.31 ± 1.09 KCl; 4.45 ± 1.88 NH_4_OAc) (LSD_KCl_ = 0.97; LSD_NH4OAc_ = 1.53). The water‐soluble Al ranged from 0.85 to 7.85 cmol kg^−1^ in all three sites with significantly high values at S3 (5.51 ± 0.82; LSD_WS‐Al _= 0.18; *p* < 0.001).

### 3.4 Soil microbial quality assessment: Microbial biomass carbon and enzymatic activity, respiration quotient, microbial metabolic quotient

The MBC content varied from 79.14 to 711.06 mg kg^−1^. It was significantly high in the S3 (603.24 ± 74.09 mg kg^−1^) compared to S2 (284.62 ± 45.29 mg kg^−1^) and S1 (118.18 ± 25.45 mg kg^−1^) (*p* < 0.001; LSD_MBC_ = 16.52) (Figure S2a). The significant positive correlation with OC (*r* = 0.94, *p* < 0.001) affirms a strong contribution of OC toward MBC. Likewise, enzyme activities vary significantly among three different sites. The hydrolytic activity of soil microbes can be represented by the nonspecific test known as FDA activity, which was, likewise, the highest in S3 (56.52 ± 8.46 µg g^−1^ h^−1^), followed by S2 (35.65 ± 6.52 µg g^−1^ h^−1^) and S1 (8.33 ± 1.49 µg g^−1^ h^−1^) (LSD_FDA _= 1.96) (Figure S2b). Other nutrient cycles (C, N, P, and S) enzymes, namely, GSD, URS, AP, AS, and DHG, are depicted in Figure S2c–g, were significantly high at S3 (*p* < 0.001), except catalase, which was contrastingly highest at S1 (Figure S2h). The mean catalase activity of S1 was 78.29 ± 4.67 mL KMnO_4_ g^−1^ soil h^−1^, followed by S2 (60.93 ± 4.98 mL KMnO_4_ g^−1^ soil h^−1^) and S3 (48.87 ± 6.21 mL KMnO_4_ g^−1^ soil h^−1^). Microbial qCO_2_ and RQ are two measurements of various perturbations in the soil ecosystem. A maximum RQ was generated for S1 (0.41 ± 0.13), while S2 (0.35 ± 0.10) and S3 (0.28 ± 0.05) showed a lower RQ (Figure S2i). Correspondingly, the qCO_2_ was higher at S1 (0.006 ± 0.002), followed by S2 (0.004 ± 0.001) and S3 (0.002 ± 0.000) (Figure S2j).

### 3.5 Solubility dynamics of ions and TCLP test

Both metals and nonmetals were found to have greater solubility at S1 compared to S2 and S3 (Figure S3a–r). The native pH of the three sites was acidic (4.0–5.1). However, at the end of incubation, pH was reduced in all sites, and the reduction was much higher at S1. Reduction of the base‐forming cation (Ca^2+^, Na^+^, Mg^2+^, and K^+^) with a corresponding increase in anionic species (SO^4−^ and Cl^−^) was exhibited at S1 with increasing time (from 7 to 21 days). SO_4_^−^ and Cl^−^ both significantly dropped in S2 and S3 (*p* < 0.001) with increasing days. However, NO_3_^−^ and PO_4_^3−^ solubility reduced in all three sites from 7 to 21 days. Al, the predominant acidic cation, showed an increasing trend of solubility at S1 till the end of incubation (*p* < 0.001). There was a significant temporal variation in PTM's solubility across the sites. Throughout the incubation period, the solubility of Mn and Zn increased with time (from 7 to 21 days) in all sites (*p* < 0.001). Overall, Pb, Cd, and Ni concentrations showed increasing trends of solubility till the end of incubation at S1, while Cu and Cr solubility declined with time (*p* < 0.001). S2 showed a significant decrease in Ni, Cr, and Pb (*p* < 0.001) with an increase in Mn and Zn content over time. At S3, Ni, Cu, and Al decreased (*p* < 0.001), but Zn, Cd, and Pb solubility considerably increased after 21 days of incubation. A slight decrease in Cr solubility was also observed at S3. Fe concentration was exceptionally high at S1 compared to S2 and S3; nevertheless, its solubility was stabilized (no significant increases or decreases in solubility) at the end of incubation. The TCLP assessment revealed that S1 had high leachability of Cu (0.56 ± 0.20), Ni (3.9 ± 1.4 mg kg^−1^), Cr (1.76 ± 0.88), and Zn (1.66 ± 0.55 mg kg^−1^), while S3 showed pronounced leaching of Pb (8.48 ± 2.06 mg kg^−1^), Cd (0.22 ± 0.80 mg kg^−1^), and Fe (9.51 ± 1.97 mg kg^−1^). Mn leachability was highest at S2 (22.69 ± 7.44 mg kg^−1^) (Figure S4a).

### 3.6 Risk assessment

A high PI (>1) of 1.48 of the S1 indicated an extensive gross metal concentration followed by S2 (1.33) and S3 (1.44) (Figure S4b). The *I*_geo_ values of the studied sites come under seven geo‐accumulation categories, where S1 showed moderate to very high contamination, followed by S2 and S3 (Figure S4c). MF calculation depicted the highest mobility rate of Cd in all three soils, followed by Ni, Pb, and Cr (Figure S4d). Table S4 represents metal‐oriented non‐cancer and cancer risk in human subjects, including children and adults. The HI values for all metals were <1 in adults, while in children, both HQ and HI were >1 for Pb. The maximum HI was recorded at S1 for children for the metal Pb (1.70), although it was high in all three sites. The cancer risk was primarily developed for Ni. The risk crossed the safer limit in both children and adults bearing the values 1.41E‐04 and 2.75E‐04, respectively, for S1, followed by S2 and S3, while for other metals (Cr, Pb, and Cd), the risk was within the safe range.

### 3.7 Similarity percentage analysis, cluster analysis, and PCA

ANOSIM and PERMANOVA analyses spotted a highly significant difference in the enzyme compositions of the studied area (*p* < 0.0001; *R*_ANOSIM_ = 0.81). The overall functional dissimilarity between the three sites evaluated according to the Bray‐Curtis dissimilarity index was 34.8% (Table 1). The highest contribution of 78.86% was recorded in the AP suggesting it as the most influential enzyme that contributes to the observed variation among locations. Additionally, GSD and URS were the second and third most influential enzymes providing 6.15% and 4.98%, respectively, and cumulatively contributed 90% to the dissimilarity matrix (Table 1). Hierarchical cluster analysis comprehended two clusters that consisted of all the enzymatic activities, acidity‐Al fractions, OC, and pH in one cluster, while all the metal fractions and catalase activity were in another cluster (Figure S5a). Three principal components, PC1, PC2, and PC3, were identified by PCA. The three PCs accounted for 91.75% of the variation, with PC1–PC3 showing the greatest decrease in variation (Figure S5b). The first principal factor (PC1) alone described 84.04% of the total variance of the dataset, while the PC2 and PC3 accounted for 6.15% and 1.57%, respectively. Based on the extracted eigenvectors, PC1 accounted for all microbiological properties, including acidity fraction, Al, CEC, and OC, whereas PC2 and PC3 accounted for metal fractions and pH.

**TABLE 1:** The similarity percentage (SIMPER) analysis of enzyme activities contributing to the functional dissimilarity between sites in the Bray–Curtis dissimilarity matrix.

Enzyme	Overall average dissimilarity 34.80%					
	Average. dissimilarity	Contribution %	Cumulative %	Mean S1	Mean S2	Mean S3
Acid phosphatase	27.44	78.86	78.86	443	753	1.3E03
β‐D‐glucosidase	2.14	6.15	85.02	11.4	42.5	75.7
Urease	1.73	4.98	90	23.3	43	77.2
Fluorescein diacetate (FDA) activity	1.64	4.71	94.72	8.33	35.7	56.5
Catalase	1.01	2.90	97.62	78.3	60.9	48.9
Dehydrogenase	0.46	1.32	98.95	1.49	7.08	15.6
Aryl sulfatase	0.36	1.04	100	2.2	6.22	13.6

*Note*: S1: mining waste dumped soil [MWDS]; S2: reclaimed site soil [RSS]; and S3: soil from the site with sparse vegetation [SSV].

## 4 DISCUSSION

### 4.1 Physicochemical and microbiological properties of the soil

The current investigation uncovered the underlying geochemistry of the bauxite mine impacted soil and its significance on soil microbiota. Three study sites (S1, S2, and S3) were widely diversified in terms of their physicochemical and biological properties. The lower pH of all sites demonstrated the acidic property of the mining‐affected soil. Given that bauxite is the raw material used to extract Al, the presence of Al^3+^ ions can be understood as a primary contributing factor to soil acidity. Previously, Sahoo et al. (2010) reported on such Al‐induced acidity in coal mining areas. However, Dolui and Mondal (2007) suggested that different forms of Fe and Al predominantly govern the acidity of acid soil, which aligns with our result. Nevertheless, since Al^3+^ can hydrolyze and release the H^+^ ion into the soil solution, it is also possible that the H^+^ ion is responsible for the decrease in pH (Sanyal & Bhattacharyya, 2012). Under such acidic conditions, base‐forming ions are likely replaced by H^+^ and Al^3+^ ions through cation exchange (Goulding, 2016). However, the organic fractions of soil tend to facilitate the high CEC of S3, which is likely due to its clay texture and high OM that efficiently adsorb the positively charged cations. TOC and OM were highest at S3, which had the highest CEC. Such high OM with greater CEC under acidic pH could be due to the dissociation of H^+^ from the carboxyl group in humic constituents (Johnson, 2002). Mineralizable N was significantly high at S3 due to OM deposition from the site's vegetation. It is interesting to note that the study region with the highest OC content (S3) showed significantly higher acidity compared to S2 and S1. Such high acidity mostly comes from TPA and PDA. Although TPA sums up all fractions of acidity, PDA has higher pH‐dependent charges, which are associated with higher Fe and Al‐oxide concentrations accompanied by a high amount of OM (Sanyal, 1991). There was also a strong positive correlation seen between ExA and Al (*r* = 0.91, *p* < 0.001), and the high availability of Ex‐Al ions implies a major role of Al ions in boosting soil acidity in such scenarios. Recently, Sulakhudin et al. (2023) stated that Al is one of the governing factors on generation acidity in bauxite mine‐contaminated land. In line with the study of Pal et al. (2007) and Sahoo et al. (2010), the Ext‐Al (KCl and NH_4_OAc) was significantly high in the organically rich site (i.e., S3), supposed to form a stable organo‐Al complex (LSD_KCl_ = 0.97; LSD_NH4OAc _= 1.53). This is further supported by a strong positive correlation with TOC (*r*_KCl_ = 0.87, *r*_NH4OAc_ = 0.90, *p* < 0.001). However, the extent of OM decomposition and the probable presence of dissolved OM potentially influence the availability of Al as well as other metals in the studied area.

The presence of metals in mine‐affected soil is widely documented in the literature (Banerjee et al., 2023; Zhong et al., 2020). However, there is limited research focusing specifically on metals and metalloids in bauxite mines (Kusin et al., 2018; Rezaei et al., 2019). In contrast, our study identified a significant presence of PTMs at all three sites (S1, S2, and S3). Although metal exists in different fractions in the soil matrix, nevertheless, the significant presence in the labile pool (i.e., WS and Ex fraction) at S1 is a concern, as they are potentially bioavailable and pose a significant impact on soil microbial activity. Lower pH often results in increased metal solubility owing to decreased metal sorption onto the soil particles (Zhong et al., 2020). The studied area had a lower pH (<5), resulting in a substantial presence of PTMs like Cd, Pb, Ni, Cr, and Cu in three fractions (WS, Ex, and CBD) following the mineral‐bound (i.e., RS) fraction. Although the CBD fraction is not easily accessible to soil microorganisms, yet they are weakly bound and can easily shift through a chemical equilibrium mechanism into a bioavailable (i.e., Ex) form that may lead to hazards to soil ecophysiology in the long run (Goswami et al., 2016). Since TOC is an important regulatory factor of metal availability due to the formation of organometal complexes, their poor availability in the S1 soil suggests the metal exists in mobile form. Consequently, such elevated metal occurrence significantly lowered the microbial activity in the S1 soil (*p* < 0.001). In contrast, despite high acidity, the S3 soil is organically rich, thereby microbially enriched. While the toxicity and availability of metals are influenced by soil pH and acidity, as in other studies (Chakraborty et al., 2024; Ghosh et al., 2023), the microbial activity of the studied area purely depends upon OC and OM availability, which perhaps immobilizes metal ions and provides energy for microbial growth, proliferation, and activity.

Microbial‐derived carbon (MBC) demonstrates a high sensitivity to heavy metal concentrations, resulting in markedly reduced activity at site S1 (LSD_MBC_ = 16.52, *p* < 0.001). Furthermore, the significant positive correlation observed between metal availability and both RQ and qCO_2_ suggests that soil disturbances are a direct result of increased heavy metal concentrations. Both were significantly high at S1 compared to S2 and S3 (LSD_RQ _= 0.03; LSD_qCO2_ = 0.0004; *p* < 0.001). In such OM‐deprived soil, increased qCO_2_ delineates the energy shifting from microbial growth to maintenance, while the high RQ suggests suppression of metabolically active (zymogenous) population to dormant ones (autochthonous) (Pal et al., 2007; Sahoo et al., 2010). Likewise, all enzyme activities were significantly low at S1 due to metal ion interference, which inactivates enzymes via sulfhydryl groups except catalase (*p* < 0.001). Metals frequently chelate with substrates or enzyme–substrate complexes. Moreover, energy can be diverted to combat metal stress by synthesizing intra‐ or extracellular sequestering proteins or conducting biochemical reactions to precipitate the metal (Hu et al., 2014; F.‐P. Zhang et al., 2010). In addition, the elevated catalase activity at S1 suggests that the microbial cells are being protected from oxidative stress induced by PTM, as catalase is crucial in mitigating reactive oxygen species (ROS) (Paul et al., 2021; Wahsha et al., 2017). Such enhanced catalase activity may be the indication of microbial response to oxidative stress, which typically leads to the production of hydrogen peroxide, superoxide radicals, and singlet oxygen, collectively referred to as ROS (Verma & Dubey, 2003). In response, catalase interacts with peroxide, thereby helping the microbes to survive under stressful conditions. Earlier Wu et al. (2021) reported on high catalase activity in heavy metal‐contaminated soil. On the other hand, AP is the only enzyme that exhibited abundant presence regardless of pH; however, was also influenced by OM. The high production of AP could be attributed to the acquisition of P from organic sources in acid soil with limited P availability (Kunito et al., 2016). In contrast, high OM is plausible for the greater microbial activity at S3.

### 4.2 Dissolution precipitation dynamics, TCLP, and risk assessment

From the solubility study, it was observed that the dissolution pattern of different ions in the soil is largely regulated by pH, OM content, electrical conductivity, and so forth. In the present study, the decline of pH at S1 with increasing days is attributed to the release of Al^3+^ and SO_4_^−^ ions. Generally, Al^3+^ is the predominant acidic cation at pH < 5, which is responsible for pH reduction of leachate. Overall, S1 had much higher metal and nonmetal solubility than S2 and S3 (*p* < 0.001), which may be attributable to the site's low OM concentration. However, their higher release at S1 may be due to highly acidic pH (pH 4.68). Such acidified condition triggers the release of cations from soil Ex sites (Haynes & Swift, 1986). The temporal increment of SO_4_^−^ and Cl^−^ at S1 was a significant concern. Increased sulfur concentration may lead to severe effects on vegetation and diversity. Concurrently, excessive Cl^−^ in the environment can reduce soil fertility and cause crop toxicities, limiting agricultural land utilization. Mn and Zn concentrations increased throughout the incubation period, possibly due to fluctuating redox potential (Sahariah et al., 2015). However, the increasing concentration of Pb may be due to the formation of soluble PbCl^+^/PbCl_2_. Similarly, Cd rise may be due to the formation of CdCl^+^/CdCl_2_ under acidic conditions (Sahariah et al., 2015; Zhang et al., 2008). Interestingly, Cr concentration decreased at the end of incubation while Fe and Al increased drastically.

The visual MINTEQ model (Table S5) predicted the saturation of Al, Fe, and Pb minerals such as gibbsite Al (OH)_3_, boehmite [γ‐AlO(OH)], alunite [KAl₃(SO₄)₂(OH)₆], variscite [AlPO₄·2(H₂O)], anglesite (PbSO_4_), chloropyromorphite [Pb_5_(PO_4_)_3_Cl], diaspore [α‐AlO(OH)], hydroxylpyromorphite (Pb_5_(PO_4_)_3_OH), vivianite [Fe_3_(PO_4_)_2_·8H_2_O], plumbgummite [PbAl_3_(PO_4_)_2_(OH)_5_·H_2_O], and so forth. Interestingly, Cr‐bearing minerals, chromite (FeCr_2_O_4_) and eskolaite (Cr_2_O_3_), were predicted at S2 and S3. Phosphate‐ and sulfate‐bearing minerals had SI > 0 suggesting oversaturation and potential precipitation. However, the mineral saturation was higher at S2 and S3. Alunite and plumbgummite were oversaturated, though saturation reduced from 7 to 21 days (Table S5). The model also showed the formation of both metal and nonmetal species, including CdCl_2_, CdSO_4_, PbCl_2_, NiCl_2_, ZnCl_2_, ZnSO_4_, MnCl_2_, CuSO_4_, FeCl_2_, FeSO_4_, and MgCl_2_, all exhibiting an SI of less than 0, which suggests its solubility over time in natural conditions. At the end of incubation, the undersaturation (SI < 0) of some minerals, like glikinite [(Zn_3_O(SO_4_)_2_); SI = −33.38], ettringite [Ca_6_Al_2_(SO_4_)_3_(OH)_12_·26H_2_O); SI = −41.04], chromic chloride [(CrCl3); SI = −39.31], alum‐K [{KAl (SO_4_)_2_·12(H_2_O)}; SI = −10.83], and cadmium hydroxide sulfate [(Cd_4_SO_4_(OH)_6_.1.5H_2_O); SI = −29.24], indicated high dissolution potential.

Based on the TCLP results, the concentrations of Cr, Cu, and Cd were within the regulatory limits, while Pb leaching exceeded the standard limit set by USEPA regulations (USEPA, 2023). The extremely low pH (2.88) of the extractant resulted in the dissolution of more metal in the leachate (Golui et al., 2020). Consequently, the rate of leaching was higher at S3, which was most likely influenced by its high acidity. Mining activities can readily lead to pollution due to many factors such as tailing waste deposition, AMD, mineral oxidation, and low biomass production (Iskandar et al., 2022). Moreover, the *I*_geo_ indicated that the studied area is moderate to heavily contaminated with Cd and Mn, while the high occurrence of Ni, Cr, and Pb designated it as high to very highly contaminated. Earlier, Equeenuddin et al. (2013) and Manna and Maiti (2018) reported the magnitude of pollution in the coal mine region due to the abundance of heavy metals. Acidic pH often causes metals to be mobile; additionally, low OM concentration promotes this mobility; as a result, S1 had high mobility for Cd > Pb > Ni > Cr > Cu > Zn > Mn > Fe.

Recurrent exposure to PTM results in carcinogenic and noncarcinogenic health risks in children and adults through different routes (ingestion, inhalation, dermal). Pb had the highest HI of 1.70 for S1, followed by 1.39 at S2, while HI was <1 for S3 soil. Over time, the accumulation of metal in soil may cause neurological and developmental disorders in children (Ferreira‐Baptista & De Miguel, 2005). In contrast, the estimated carcinogenic risks were highest for Ni, followed by Cr, Pb, and Cd. Consequently, total carcinogenic risk executed by Ni crossed the safer limit given by USEPA (safe limit 1 × 10^−4^–1 × 10^−6^) in both children and adults. Nickel is already listed as a potent carcinogen according to the International Agency for Research on Cancer (Kabir et al., 2022); thus, constant exposure to Ni may have detrimental effects on human health.

### 4.3 Availability of PTMs–acidity–aluminum–organic carbon–soil enzymes interlinking: A correlation‐based insight

Aluminum pools showed a significant positive correlation (*p* < 0.001) with soil acidity (Figure 2c). The significant positive correlation between TOC and different acidity fractions indicated the important role that OM played on soil acidity, particularly the generation of R‐COOH and R‐OH groups (Chakraborty et al., 2024). On the other hand, a positive correlation (*p* < 0.001) between TOC and a different fraction of Al suggested the availability of Al in soil, which is regulated by diverse chemical interlinkage with OM. To counteract the pH reduction, inorganic and organic Al complexes often dissociate, which promotes the mobilization of inorganic monomeric Al that induces toxicity to the living cells (W. Li & Johnson, 2016). In the present study, the overall sampled site had an exceptionally low pH (∼5). Along with that, high acidity and Al persisted at S3, which also contained high TOC and OM coupled with decent microbial activity.

The enzyme activities were strongly positively correlated with TOC (*p* < 0.001), indicating enzyme activity possibly stabilized as enzyme–OM complexes (Dick, 1997). Thus, expressing enzyme activity on OC basis will help us better understand how acidity stress affects microbial activity. The ratio of TOC‐enzyme activity was strongly negatively correlated (*p* < 0.001) with different fractions of acidity and Al (Figure S6a). Elimination of the TOC from the microbial parameters depicted that soil acidity had a direct inhibitory effect on soil enzymes and microbial activity (Figure S7). Therefore, OC stabilized the negative effect of acidity, proving that sustainability under such an acidic condition is dreadfully challenging. Conversely, the S1 had the poorest microbial activity with high‐concentration labile (WS and Ex fractions) metal. The study pointed to a significant negative correlation (*p* < 0.001) between the labile fraction of metal and microbial activity (Figure S6b), indicating its deleterious impact, while the other fractions like OXD, CBD, and RS were not very impactful. In acidic environments, the effective toxicity of heavy metals tends to be higher than indicated by total metal concentration alone, since reduced pH results in an increased proportion of toxic, free ionic forms of metals (Pennanen, 2001). Additionally, pH influences metals complexation with organic and inorganic components, and these complexed forms are less toxic than their free ionic counterparts (Babich & Stotzky, 1985; McBride et al., 1997). As a result, soils with a higher labile fraction and lower OC content exhibited reduced microbial activity.

### 4.4 Enzyme dissimilarity, cluster analysis, and principal component analysis

According to similarity percentage (SIMPER) analysis, the AP had the greatest influence on overall enzyme dissimilarity and was identified as the most influential enzyme among the sites. As such, AP was significantly abundant across the area compared to other enzymes. Acidic soil often suffers from P limitation; therefore, in response to P scarcity, microorganisms increase the expression of the gene encoding phosphatase secretions (Zheng et al., 2019). ANOSIM and PERMANOVA analyses further supported our finding of significant differences in enzyme availability across S1, S2, and S3 (*p* < 0.0001; *R*_ANOSIM_ = 0.81). The cluster analysis elucidated that the catalase synthesis was regulated by available metal, while another cluster provided the definite relation of TOC with the microbial activity of soil despite acidity and Al. Furthermore, the presence of pH in this cluster indicated the potential influence of acidity‐Al on its declining nature. In general, soil OM contributes acidity and Al content by providing several functional groups containing H^+^ and can also form an organo‐aluminum complex (Sahoo et al., 2010). PCA further explained the relationship among microbial activity enhanced by the support of TOC despite acidity and Al at S3, while S1 had poor microbial activity owing to high metal and deficit of OM. In addition to TOC availability, the microbial abundance of S3 could also be the result of an altered microbial community resistant to Al and acidity as reported by Kunito et al. (2011) and Lewis et al. (2018).

## 5 CONCLUSION

This study provided chemical and biological analyses of the soilfrom the bauxite mine area. It identified the synergistic stressors (acidity and heavy metal) collaterally impacting the soil eco‐physiological health of the sites; however, OC/OM managed to alleviate the acidity stress and save the microbial functionality. The notable enzymatic activity and microbial biomass observed despite acidity and Al illustrate an antagonistic phenomenon where OC promotes microbial functionality and acidity‐Al together inhibit microbial growth. Such inconsistency was verified when excluding the OC effect, presenting the actual scenario of detrimental effects of acidity and Al on soil microbial properties, which are mitigated by the presence of TOC. Metals that were extractable in the labile fraction were negatively correlated to microbial properties impacting the microbial survivability at the waste‐dumped site, that is, S1. Leaching dynamics revealed that SO_4_^2−^, Cl^−^, Al, Cd, Ni, Mn, and Pb were in leachate. Overall, the sites had high ecological hazards with significant health risks to humans. This study offers a benchmark for a comprehensive examination of soil quality that could support ecosystem restoration.

## AUTHOR CONTRIBUTIONS

**Kasturi Charan**: Data curation; formal analysis; investigation; methodology; software. **Sonali Banerjee**: Formal analysis; investigation; methodology. **Jajati Mandal**: Conceptualization; resources; software; supervision; writing—review and editing. **Pradip Bhattacharyya**: Conceptualization; project administration; resources; supervision; visualization; writing—review and editing.

## CONFLICT OF INTEREST STATEMENT

The authors declare no conflicts of interest.

## Supporting information



Supporting Information
