# Supplementary material for: Soil stressors on ecophysiology of bauxite mine impacted soil: Heavy metal–acidity–organic matter nexus
Source: J Environ Qual. 2025 Jan 7;54(2):435–49. doi: 10.1002/jeq2.20666 (PMC11893285; doi:10.1002/jeq2.20666)
Supplement: Supplementary file 1 — Supporting Information [file JEQ2-54-435-s001.docx]

**Soil stressors on ecophysiology of bauxite mine impacted soil: heavy metal-acidity- organic matter nexus.**

*Kasturi Charan^a^, Sonali Banerjee^a^, Jajati Mandal^b*^, Pradip Bhattacharyya^a*^*

***^a^****Agricultural and Ecological Research Unit, Indian Statistical Institute, Giridih, Jharkhand, 815301, India*

*^b^School of Science, Engineering & Environment, University of Salford, Manchester, M5 4WT, UK*

*^*^Corresponding author:* [*J.Mandal2@salford.ac.uk*](mailto:J.Mandal2@salford.ac.uk) *,* [*pradip.bhattacharyya@gmail.com*](mailto:pradip.bhattacharyya@gmail.com)

ORCiD:

Kasturi Charan: 0000-0003-0727-4664

Sonali Banerjee: 0000-0002-5978-1732

Jajati Mandal: 0000-0003-0814-0369

Pradip Bhattacharyya: 0000-0002-9741-3180

**Table S1: Equations for risk assessment**

| **Formula** | **Remark** | **Reference** |
| --- | --- | --- |
| Pollution load index (PI) = $\frac{\frac{Cd}{3}+\frac{Cr}{100}+\frac{Cu}{100}+\frac{Ni}{100}+\frac{Mn}{300}+\frac{Pb}{100}+\frac{Zn}{300}}{7}$ | PI >1 indicates significantly high contamination potential. | Banerjee al., 2023 |
| Geo-accumulation Index (I_geo_) _=_ log_2_$\frac{Cn}{1.5Bn}$ | Where C_n_ indicates the concentration of any metal, B_n_ is the concentration of a similar metal in the adjacent soil, and the constant 1.5 is used to study the natural discrepancy of the element in the sample related to its anthropogenic history. The I_geo_ values have been categorized into seven classes of contamination levels are provided in Table S2. | Mondal et al., 2017 |
| Mobility factor (MF) % = $\frac{F1+F2+F3}{\sum Fractions}$ |  | Kabala and Singh, 2001 |
| Hazard quotient (HQ) =$\frac{Intakes (Ingestion or Inhalation or Dermal)}{\mathrm{RfD}}$  Hazard index (HI) = ∑ HQexP | The exP denotes different exposure pathways (Intake_ingestion_, Intake_inhalation,_ and Intake_dermal_). While the R_f_D indicates the limit of exposure to the pollutant which is supposed to be harmless during a lifetime. | Mondal et al., 2017 |
| Cancer risk (CR) = ADD x SF_i_  Total cancer risk (TCR) = ∑CR | Where ADD is the average daily intake (ingestion/ inhalation/ dermal) and SF_i_ is the cancer slope factor (mg kg^-1^day^-1^). CR value ranges from 1x10^-6^ to 1x10^-4^ and is harmless for human health ones the value exceeds 1×10^−4^, it represents a lifetime carcinogenic risk to the human body. | USEPA, 2002 and Banerjee et al., 2023 |

**Table S2: Classification of contamination level based on I_geo_ values (Muller, 1969).**

| **I_geo_ Classes** | **Contamination level** |
| --- | --- |
| I_geo_ ≤ 0; Class 0 | uncontaminated |
| I_geo_0–1; Class 1 | uncontaminated to moderately contaminated |
| I_geo_1–2; Class 2 | moderately contaminated |
| I_geo_2–3; Class 3 | moderately to highly contaminated |
| I_geo_3–4; Class 4 | highly contaminated |
| I_geo_4–5; Class 5 | highly to very highly contaminated |
| I_geo_ ≥ 5; Class 6 | very highly contaminated |

**Table S3: Basic properties of the study area. (S1 - Mining waste dumped soil, MWDS; S2 - Reclaimed site soil, RSS; S3 - Soil from the site with sparse vegetation, SSV). Data represented as mean±SD; LSD-least significant difference.**

| **Parameters** | **S1** | **S2** | **S3** | **p-value** | **LSD** |
| --- | --- | --- | --- | --- | --- |
| pH | 4.33±0.30 | 5.09±0.17 | 4.54±0.28 | < 0.001 | 0.08 |
| TOC (%) | 0.15±0.03 | 0.91±0.31 | 1.93±0.15 | < 0.001 | 0.06 |
| Organic Matter (%) | 0.27±0.06 | 1.57±0.53 | 3.40±0.27 | <0.001 | 0.11 |
| Mineralizable N (%) | 0.002±0.0009 | 0.006±0.002 | 0.011±0.005 | < 0.001 | 0.001 |
| Sand (%) | 71.43±9.55 | 53.56±9.02 | 23.83±3.59 | <0.001 | 8.25 |
| Silt (%) | 7.20±1.83 | 27.14±3.22 | 24.90±4.05 | <0.001 | 3.75 |
| Clay (%) | 21.38±3.26 | 18.87±2.11 | 35.56±5.03 | <0.001 | 4.49 |
| Exchangeable Ca^2+^ (mg kg^-1^) | 10.21±4.27 | 485.61±114.21 | 141.48±27.33 | <0.001 | 21.45 |
| Exchangeable K^+^ (mg kg^-1^) | 10.26±3.55 | 140.37±32.10 | 100.61±16.96 | <0.001 | 6.66 |
| Exchangeable Mg^2+^ (mg kg^-1^) | 22.88±9.16 | 1139.74±207.36 | 66.07±9.08 | <0.001 | 37.93 |
| Exchangeable Na^+^ (mg kg^-1^) | 14.32±3.28 | 33.64±7.9 | 23.18±6.76 | <0.001 | 1.99 |
| Cation exchange capacity (CEC) (cmol kg^-1^) | 5.42±1.53 | 10.59±1.51 | 24.45±3.75 | <0.001 | 0.79 |
| Available P (mg kg^-1^) | 4.68±1.44 | 7.93±2.71 | 5.74±1.26 | < 0.001 | 0.60 |
| Total Cu (mg kg^-1^) | 91.04±10.17 | 64.02±8.04 | 51.33±4.33 | <0.001 | 3.87 |
| Total Ni (mg kg^-1^) | 108.42±8.92 | 114.05±11.82 | 103.95±13.82 | <0.001 | 6.70 |
| Total Cr (mg kg^-1^) | 79.72±11.62 | 72.27±13.11 | 45.70±8.25 | <0.001 | 5.41 |
| Total Pb (mg kg^-1^) | 145.79±11.32 | 129.75±6.30 | 91.19±14.19 | <0.001 | 8.50 |
| Total Cd (mg kg^-1^) | 9.57±1.18 | 5.97±1.14 | 3.39±1.52 | <0.001 | 1.44 |
| Total Zn (mg kg^-1^) | 68.06±8.86 | 58.98±4.7 | 64.45±5.78 | 0.003 | 2.07 |
| Total Mn (mg kg^-1^) | 152.93±13.01 | 416.90±68.81 | 1481.54±209.32 | <0.001 | 40.06 |
| Total Fe (mg kg^-1^) | 159316.3±43679.62 | 36297.83±7110.65 | 40307.38±3258.80 | <0.001 | 8101.62 |

**Table S4: Non-carcinogenic (three exposure pathways) and carcinogenic risks for adults and children from mine impacted soil.**

| Sample ID | Metal | Adult | | | |  | Child | | | |  |
| --- | --- | --- | --- | --- | --- | --- | --- | --- | --- | --- | --- |
|  |  | Hazard Quotient (HQ) | | | Hazard index (HI) | Total cancer risk (TCR) | Hazard Quotient (HQ) | | | Hazard index (HI) | Total cancer risk (TCR) |
|  |  | Ingestion | Inhalation | Dermal |  |  | Ingestion | Inhalation | Dermal |  |  |
| S1 | Ni | 0.011010 | 1.57E-06 | 0.0001627 | 0.0112 | 1.41E-04 | 0.08807705 | 0.0002394 | 0.0009134 | 0.0892 | 2.75E-04 |
|  | Cd | 0.017064 | 2.51E-06 | 0.0068085 | 0.0239 | 2.93E-06 | 0.13651137 | 3.814E-06 | 0.0382232 | 0.1747 | 5.85E-06 |
|  | Cr | 0.072140 | 1.11E-03 | 0.0143919 | 0.0876 | 4.36E-05 | 0.57712072 | 0.0016915 | 0.0807969 | 0.6596 | 8.28E-05 |
|  | Cu | 0.004955 | 7.29E-07 | 0.0000659 | 0.0050 |  | 0.03963775 | 1.108E-06 | 0.0003858 | 0.0400 |  |
|  | Mn | 0.005981 | 2.83E-03 | 0.0005966 | 0.0094 |  | 0.04784962 | 0.0043008 | 0.0033495 | 0.0555 |  |
|  | Pb | 0.211510 | 1.24E-05 | 0.0022548 | 0.2138 | 1.81E-05 | 1.6920792 | 1.88E-05 | 0.0126583 | **1.7048** | 2.59E-05 |
|  | Zn | 0.000692 | 1.02E-07 | 0.0000138 | 0.0007 |  | 0.00553735 | 1.547E-07 | 0.0000775 | 0.0056 |  |
| S2 | Ni | 0.009987 | 1.43E-06 | 0.0001476 | 0.0101 | 1.28E-04 | 0.07989777 | 0.0002172 | 0.0008286 | 0.0809 | 2.49E-04 |
|  | Cd | 0.011826 | 1.74E-06 | 0.0047187 | 0.0165 | 2.03E-06 | 0.09461005 | 2.644E-06 | 0.0264908 | 0.1211 | 4.05E-06 |
|  | Cr | 0.065888 | 1.02E-03 | 0.0131446 | 0.0800 | 3.98E-05 | 0.52710159 | 0.0015449 | 0.0737942 | 0.6024 | 7.57E-05 |
|  | Cu | 0.003692 | 5.43E-07 | 0.0000491 | 0.0037 |  | 0.02953352 | 8.252E-07 | 0.0002875 | 0.0298 |  |
|  | Mn | 0.016611 | 7.86E-03 | 0.0016569 | 0.0261 |  | 0.1328842 | 0.0119437 | 0.0093019 | 0.1541 |  |
|  | Pb | 0.172920 | 1.01E-05 | 0.0018434 | 0.1748 | 1.48E-05 | 1.38336386 | 1.537E-05 | 0.0103488 | **1.3937** | 2.12E-05 |
|  | Zn | 0.000557 | 8.19E-08 | 0.0000111 | 0.0006 |  | 0.0044572 | 1.245E-07 | 0.0000624 | 0.0045 |  |
| S3 | Ni | 0.008573 | 1.22E-06 | 0.0001267 | 0.0087 | 1.10E-04 | 0.06858111 | 0.0001864 | 0.0007112 | 0.0695 | 2.14E-04 |
|  | Cd | 0.006929 | 1.02E-06 | 0.0027647 | 0.0097 | 1.19E-06 | 0.0554319 | 1.549E-06 | 0.0155209 | 0.0710 | 2.38E-06 |
|  | Cr | 0.029449 | 4.54E-04 | 0.0058750 | 0.0358 | 1.78E-05 | 0.23558898 | 0.0006905 | 0.0329825 | 0.2693 | 3.38E-05 |
|  | Cu | 0.002186 | 3.21E-07 | 0.0000291 | 0.0022 |  | 0.01748603 | 4.886E-07 | 0.0001702 | 0.0177 |  |
|  | Mn | 0.054778 | 2.59E-02 | 0.0054641 | 0.0862 |  | 0.43822363 | 0.0393879 | 0.0306757 | 0.5083 |  |
|  | Pb | 0.104070 | 6.09E-06 | 0.0011094 | 0.1052 | 8.90E-06 | 0.83256399 | 9.252E-06 | 0.0062283 | **0.8388** | 1.27E-05 |
|  | Zn | 0.000495 | 7.29E-08 | 0.0000099 | 0.0005 |  | 0.00396397 | 1.108E-07 | 0.0000555 | 0.0040 |  |

| **Minerals** | **Saturation Index*** | | | | | | | | |
| --- | --- | --- | --- | --- | --- | --- | --- | --- | --- |
|  | **S1** | | | **S2** | | | **S3** | | |
|  | **7D** | **14D** | **21D** | **7D** | **14D** | **21D** | **7D** | **14D** | **21D** |
| Al(OH)_3_ (am) | 0.16 | - | - | 1.88 | 2.59 | 2.11 | 2.60 | 2.14 | 2.47 |
| Al(OH)_3_ (Soil) | 2.67 | 1.90 | 2.05 | 4.39 | 5.10 | 4.62 | 5.11 | 4.65 | 4.98 |
| Al_2_O_3_(s) | 2.29 | 0.75 | 1.05 | 5.70 | 7.12 | 6.17 | 7.15 | 6.22 | 6.89 |
| Al_4_(OH)10SO_4_(s) | 7.38 | 5.32 | 5.42 | 13.92 | 15.57 | 13.66 | 15.35 | 13.54 | 14.38 |
| AlOHSO_4_(s) | 0.42 | 0.69 | 0.34 | 1.81 | 1.34 | 0.85 | 1.08 | 0.66 | 0.49 |
| AlPO4x1.5H2O | 1.11 | 0.71 | 0.36 | 1.34 | 1.69 | 1.32 | 2.10 | 1.96 | 1.66 |
| Alunite | 7.73 | 7.30 | 6.62 | 13.05 | 13.24 | 11.21 | 12.54 | 10.93 | 11.15 |
| Anglesite | - | - | - | 0.04 | 0.09 | - | - | - | - |
| Boehmite | 2.39 | 1.62 | 1.77 | 4.10 | 4.81 | 4.33 | 4.82 | 4.36 | 4.70 |
| Chloropyromorphite(c) | 6.37 | 3.51 | 3.08 | 8.29 | 12.92 | 8.40 | 10.65 | 10.96 | 11.44 |
| Chloropyromorphite(soil) | 7.17 | 4.31 | 3.88 | 9.09 | 13.72 | 9.20 | 11.45 | 11.76 | 12.24 |
| Cr(OH)_3_ (am) |  |  |  | 0.36 | 1.00 | 0.45 | 1.33 | 0.88 | 1.19 |
| Cr2O3 (c) |  |  |  | 1.05 | 2.33 | 1.23 | 3.00 | 2.09 | 2.73 |
| Diaspore | 4.10 | 3.32 | 3.47 | 5.81 | 6.52 | 6.04 | 6.53 | 6.06 | 6.40 |
| FeCr_2_O_4_(s) |  |  |  | 0.97 | 3.13 | 1.53 | 4.54 | 3.20 | 4.24 |
| Gibbsite (C) | 3.23 | 2.45 | 2.60 | 4.94 | 5.65 | 5.17 | 5.66 | 5.20 | 5.53 |
| Hercynite | 7.89 | 5.64 | 6.07 | 10.90 | 13.20 | 11.75 | 13.97 | 12.60 | 13.69 |
| Hinsdalite | - | - | - | 0.99 | 2.81 | 0.50 | 2.31 | 1.09 | 1.50 |
| Hydroxylpyromorphite | - | - | - | - | 4.23 | - | 2.11 | 2.10 | 2.90 |
| MnHPO_4_(s) | - | - | - | - |  | 0.05 | 0.55 | 0.47 | 0.27 |
| Pb_3_(PO_4_)_2_(s) | - | - | - | - | 1.96 | - | 0.77 | 0.83 | 1.18 |
| Plumbgummite | 10.00 | 7.52 | 7.20 | 13.58 | 16.22 | 14.02 | 16.39 | 15.45 | 15.73 |
| Variscite | 2.72 | 2.31 | 1.97 | 2.95 | 3.30 | 2.93 | 3.71 | 3.57 | 3.27 |
| Vivianite | 3.59 | 2.18 | 1.59 | - | 1.42 | 0.10 | 4.40 | 3.75 | 3.72 |
| Larnakite | - | - | - | - | 0.32 | - | - | - | - |

**Table S5:** **Geochemical model (Visual MINTEQ) predicted saturation profiles of different minerals through dissolution of various cations and anions from mine impacted soil of three different sits. (S1 - Mining waste dumped soil, MWDS; S2 - Reclaimed site soil, RSS; S3 - Soil from the site with sparse vegetation, SSV).**

***Saturation Index > 1 signifies high saturation; *D- days.**

**
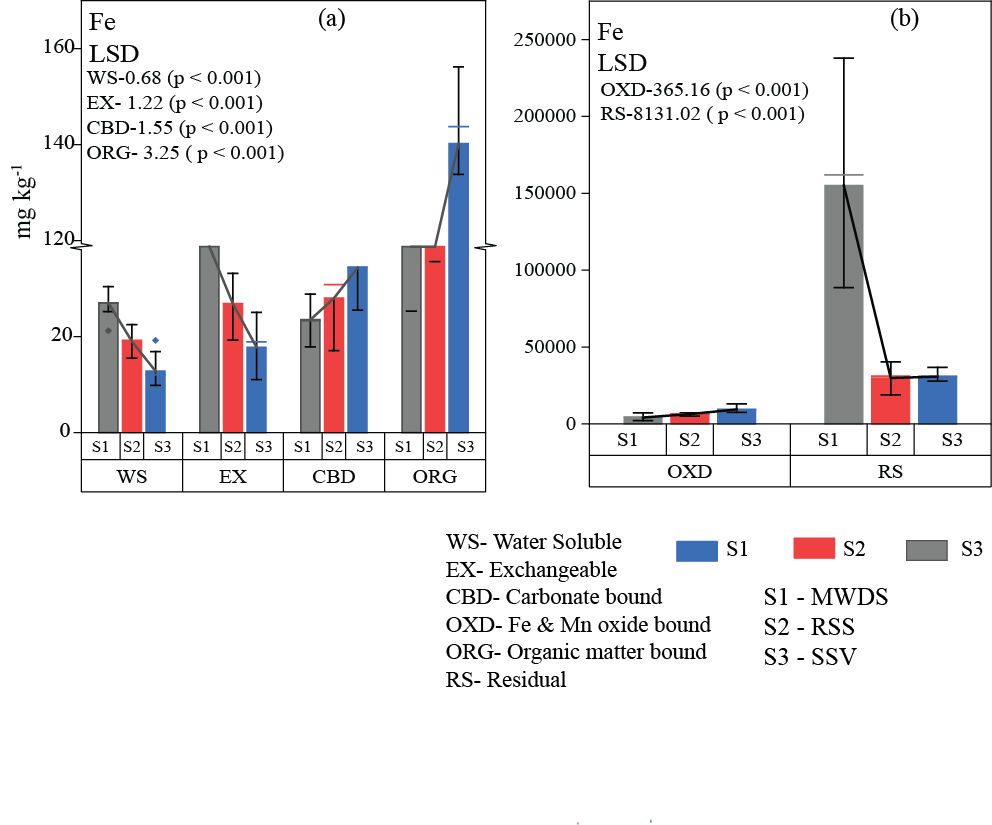
Fig. S1:** Fractional distribution of iron (Fe) at three sites of the study area. (S1 - Mining waste dumped soil, MWDS; S2 - Reclaimed site soil, RSS; S3 - Soil from the site with sparse vegetation, SSV). LSD-least significant difference.


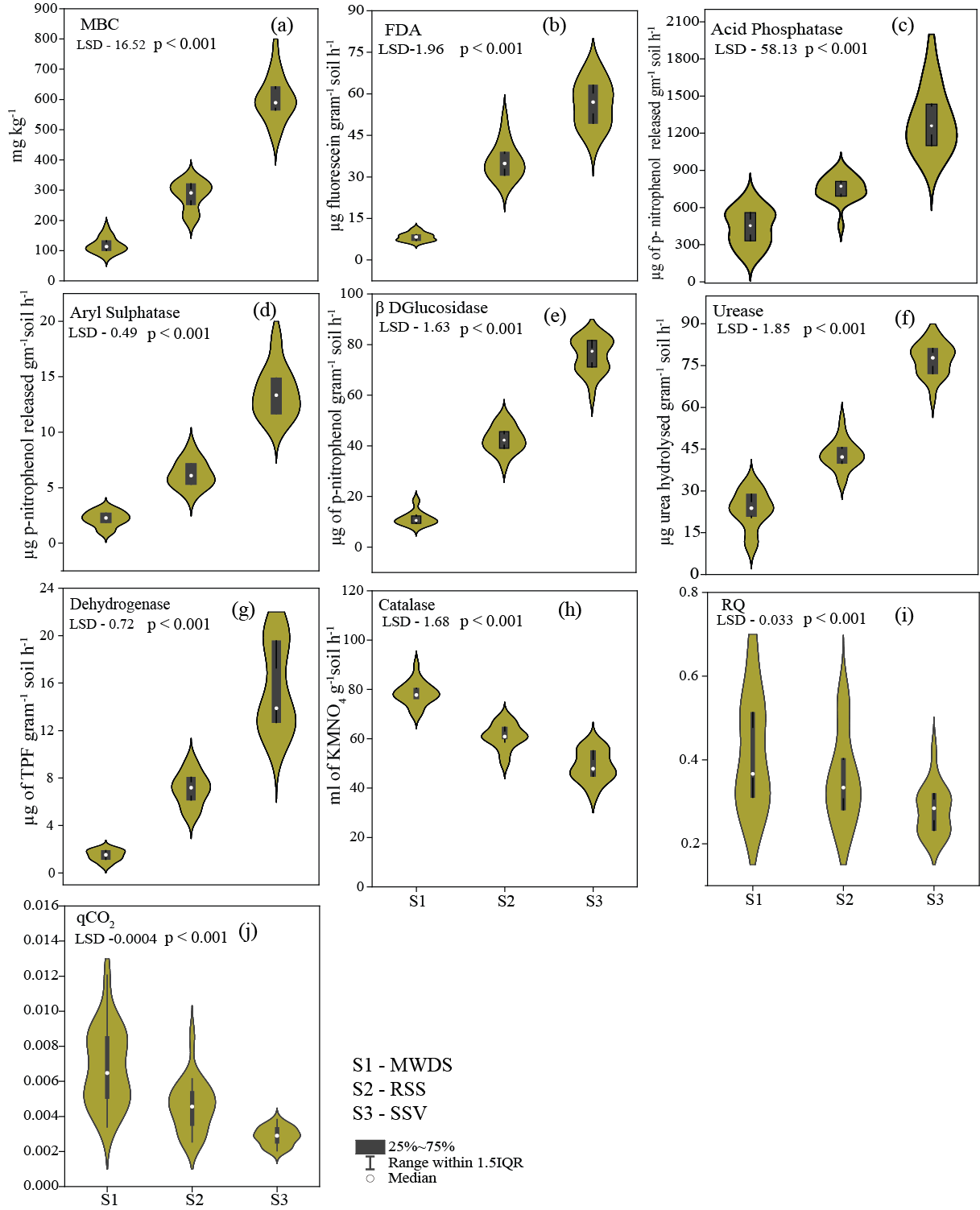


**Fig. S2:** Violin plot representing microbial and biochemical properties of three different sites. (S1 - Mining waste dumped soil, MWDS; S2 - Reclaimed site soil, RSS; S3 - Soil from the site with sparse vegetation, SSV). LSD-least significant difference.


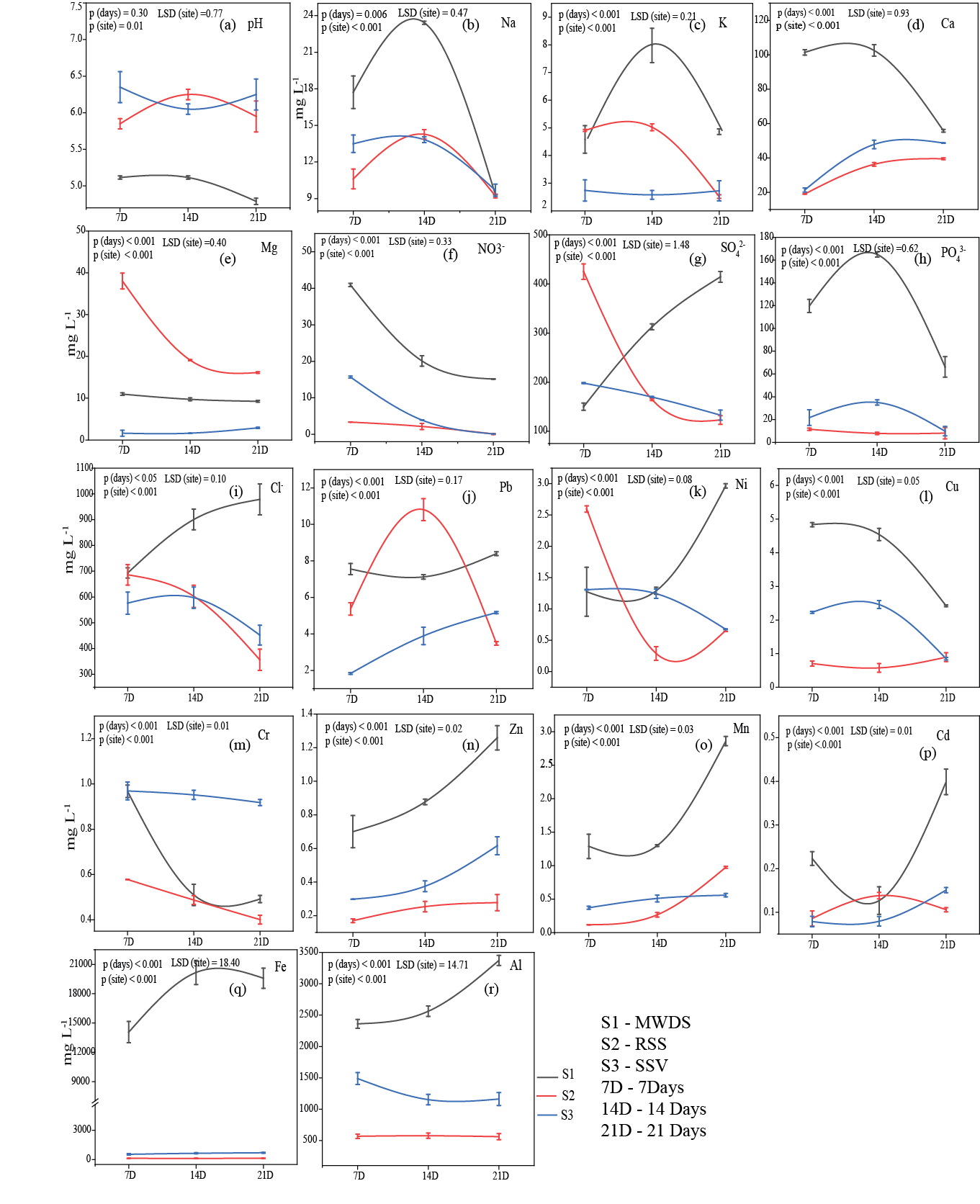


**Fig. S3:** Temporal variation of pH, cationic, anionic pollutants and potentially toxic metal (PTMs) in the water soluble phase three different sites. (S1 - Mining waste dumped soil,MWDS; S2 - Reclaimed site soil, RSS; S3 - Soil from the site with sparse vegetation, SSV). Data represented as mean±SD; LSD-least significant difference.


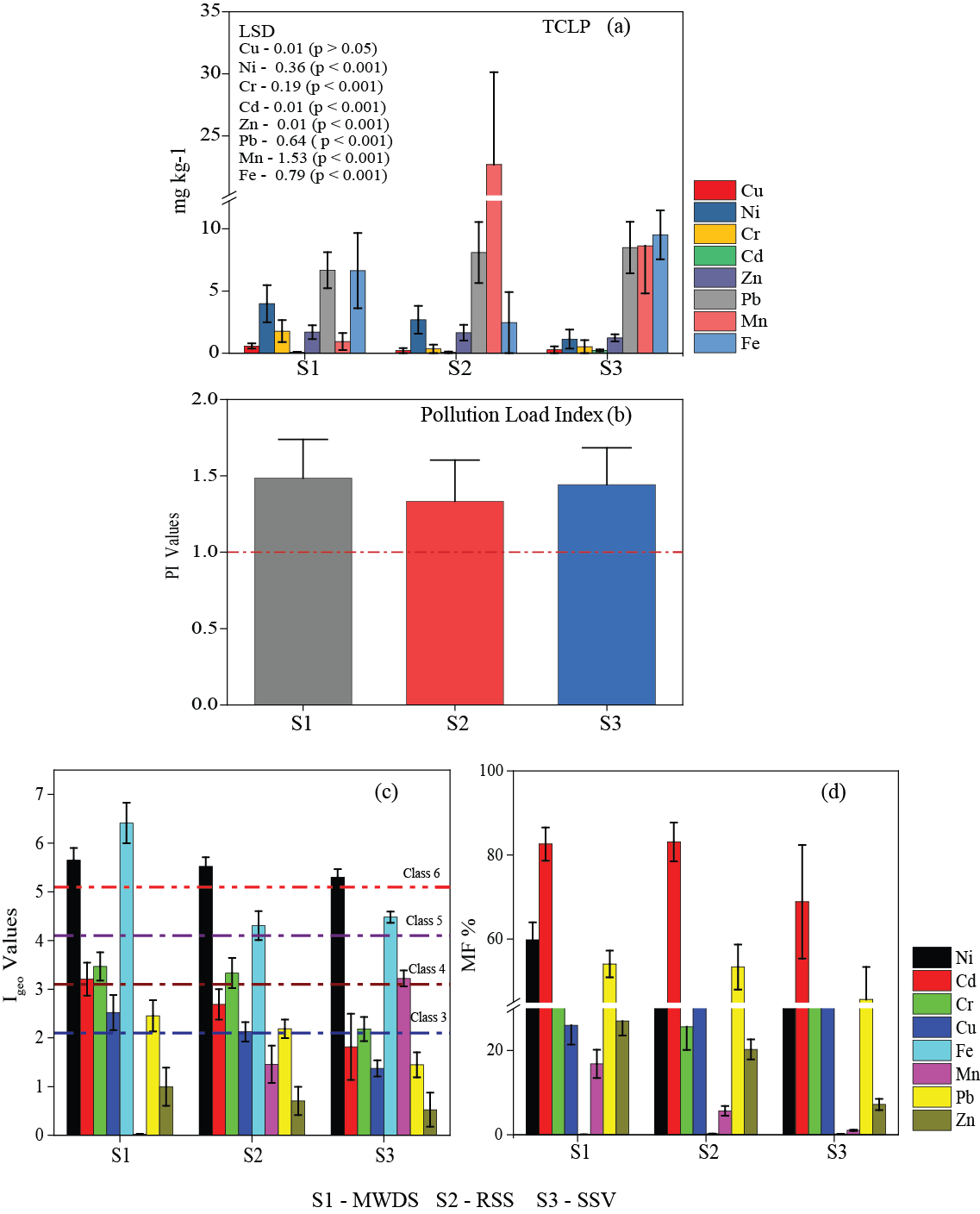


**Fig. S4**: Concentration of potentially toxic metals (PTMs) by toxicity characteristics leaching procedure at three different sites. (S1 - Mining waste dumped soil, MWDS; S2 - Reclaimed site soil, RSS; S3 - Soil from the site with sparse vegetation, SSV). Data represented as mean ± SD; LSD-least significant difference.


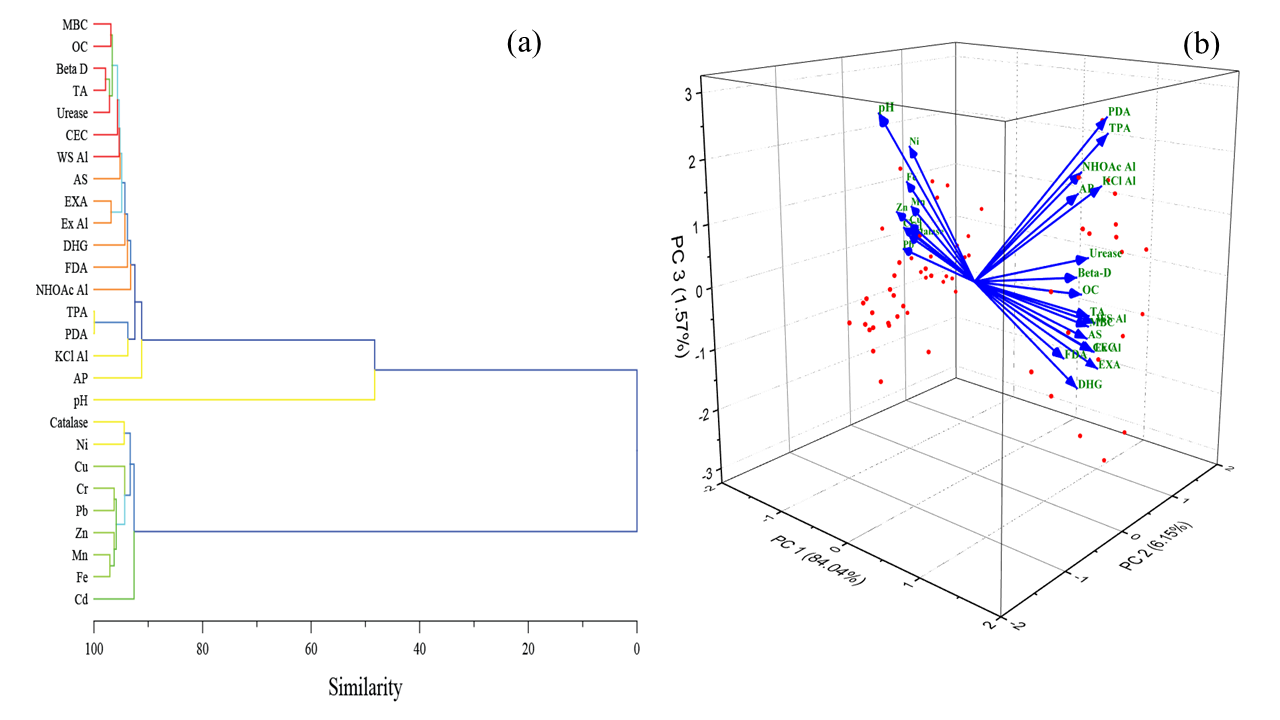


**Fig. S5:** (a) Hierarchical cluster analysis of soil physicochemical and microbial parameters. (b) Principal component analysis depicting association between physicochemical and microbial parameters.


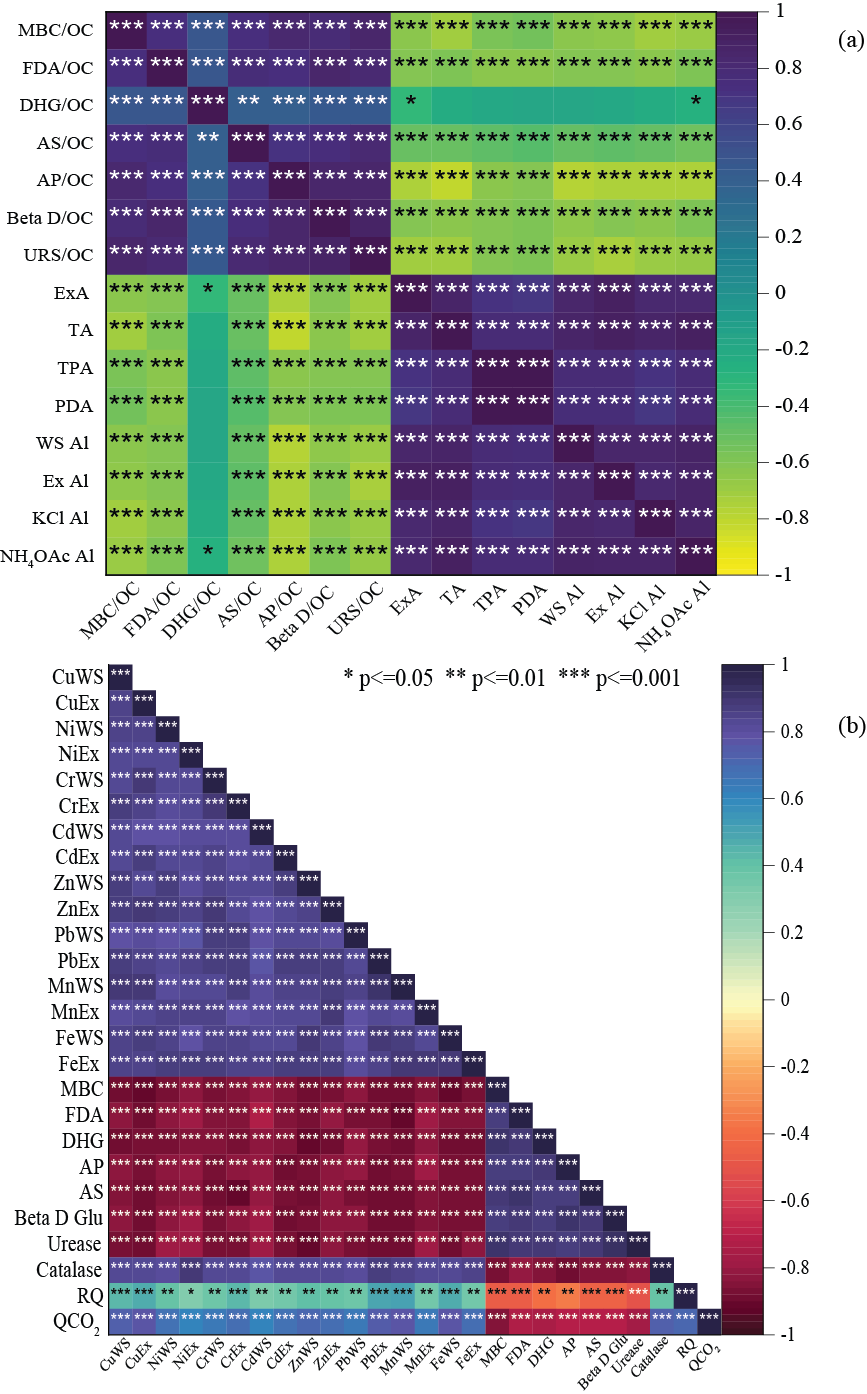


**Fig. S6:** (a) Heatmap presenting the correlation between microbial properties and different fractions of acidity and aluminum (Al) (b) Correlation plot presenting the microbial interaction with labile pool of PTMs (WS and Ex-phase). (each point indicate the correlation coefficients and their significance levels are indicated by colour of the points).


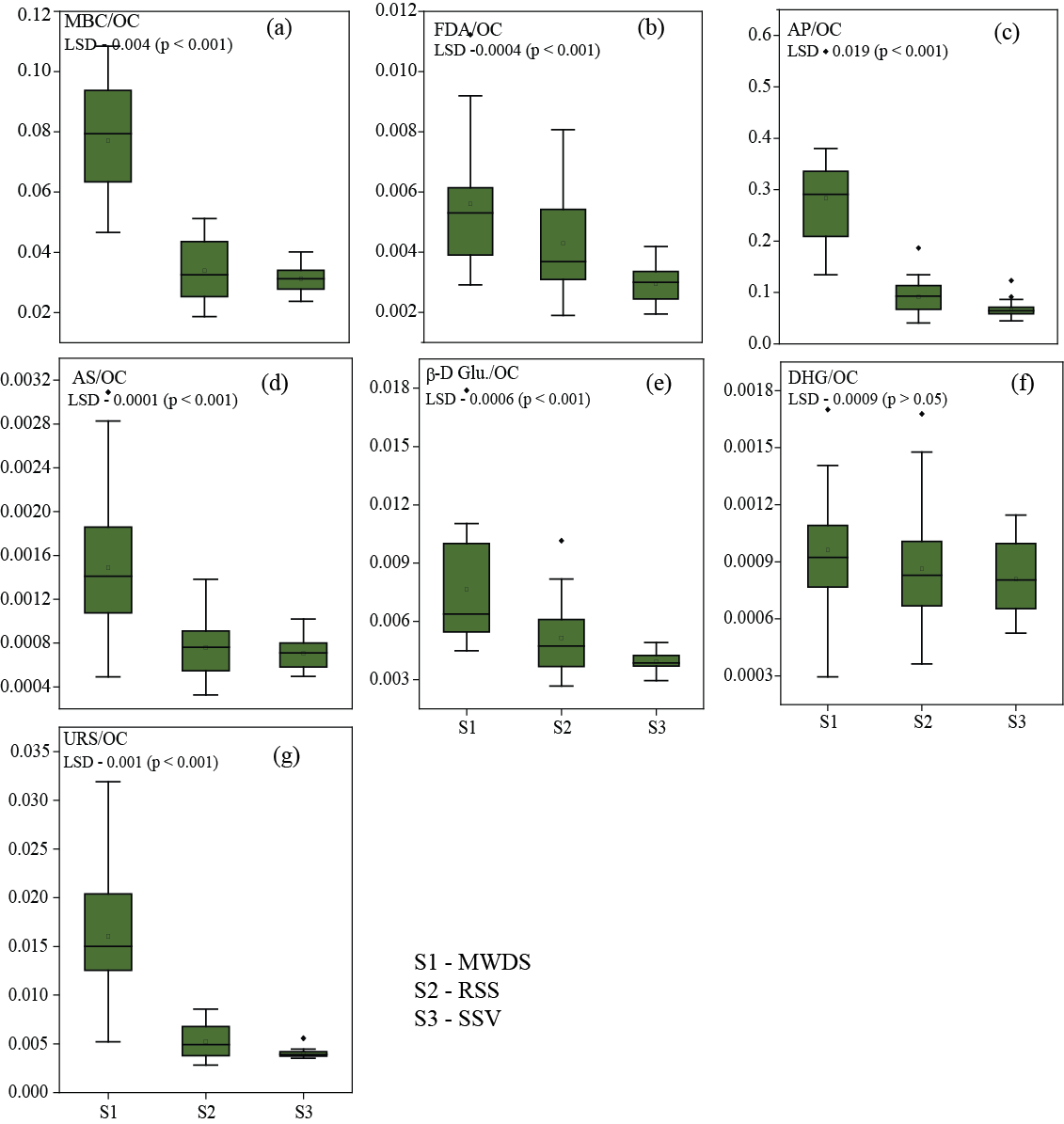


**Fig. S7:** Microbial properties/OC ratio of the three different sites. (S1 - Mining waste dumped soil, MWDS; S2 - Reclaimed site soil, RSS; S3 - Soil from the site with sparse vegetation, SSV). LSD-least significant difference.

**Supplementary Section 1:**

**Sequential extraction of various metal fractions**

The metal fractionation was studied to determine the distribution of various soluble and resistant metal forms in soil. Six separate metal fractions were extracted in accordance with Tessier et al. (1979). The following steps were taken successively to generate different fractions.:

(a) Water soluble fraction (WS, F1): 40 mL deionized water was added to 1 g sample (dry weight basis) and shaken for 30 min subsequently filtered at room temperature. The filtrate was undergone for analysis.

(b) Exchangeable fraction (Ex, F2): The residual from (a) was extracted with 1 M Mg (NO_3_)_2_ upon shaking for 30 minutes and the filtered. The filtrate was undergone for analysis.

(c) Carbonate-bound fraction (CBD, F3): The residual from (b) was extracted by shaking for five hours with 1 M NaOAc and then filtered. The filtrate was undergone for analysis.

(d) Fe and Mn oxide-bound fraction (OXD, F4): The residual from (c) was extracted by shaking with 0.08 mol/L NH_2_OH HCl as the extractant for 6 hours at 96^◦^C. After that cooled and filtered for analysis

(e) Organic matter-bound fraction (ORG, F5): A 10 mL mixture of 0.02 (M) HNO_3_ and 5 ml of 30% H_2_O_2_ was added to the residual solution from (d) and heated at 85^◦^C for 5 hours; cooled, then a second aliquot of 5 mL 3.2 (M) NH_4_OAc was added and shake for 30 minutes at room temperature, then filtered to obtain fraction.

(f) Residual-bound fraction (RS, F6): HNO_3_ (conc.) was added to the residual solution from (e) and heated (105^◦^C) till dryness and the dried residue was re-constituted in 50 mL deionized water.

All the filtrate from F1 to F6 fraction were preserved in a sterile container, and finally analysed to detect the presence of PTMs using atomic absorption spectroscopy (Systronics AA S-816).

**Supplementary Section 2**

Anions of the soil extract were measure by following the standard protocol (

*Measurement of NO_3_*^-^: NO_3_^-^ was measured with ion selective electrodes (Orion Star A214 pH/ISE Benchtop meter, Serial no:X49554, Software version: 3.04, Thermo Scientific, USA). Nitrogen Interference Supressor Solution (NISS, Orion® 930,710) and Nitrate standard of 1000 ppm (Orion®). Thermo Scientific 920,707 (USA) was utilised.

*Measurement of PO_4_^-^:* PO_4_^-^ was measured by spectrophotometric method. 5 ml of soil extract was mixed with chloromolybdic reagent. Following 1 ml of stannous chloride (SnCl_2_) was added to the mixture and finally volume make up to 50 ml. The presence of PO_4_^-^ was detected by measuring the optical density in a UV-Vis spectrophotometer at 660 nm (Systronics 117, India).

*Measurement of SO_4_^-^*: SO_4_^2-^ is the available sulphur measured using a typical turbidimetric method in a UV-Vis spectrophotometer (Systronics 117, India). 5 ml of soil extract was mixed with 10 ml of barium chloride (BaCl_2_) solution and shake for 1 minutes. Following 1 ml of 0.25% gum acacia was added to the mixture and finally volume make up to 50 ml. The presence of SO_4_^2-^ was detected by measuring the optical density in a UV-Vis spectrophotometer at 420 nm (Systronics 117, India).

*Measurement of Cl^-^*: Cl^-^ was determined using the conventional argentometric titration method, with silver nitrate (AgNO_3_) (Merck, India) and potassium chromate (Merck, India) as indicators. 5 ml of soil extract was taken, to which 5 to 6 drops of K_2_Cr_2_O_4_ indicator was added and titrated against 0.02 (N) of Ag NO_3_. Appearance of reddish brown indicated the presence of chloride in the soil extract. The amount of AgNO_3_ consumed determine the amount of chloride in the solution.
